# Finite Element Analysis (FEA) for Influence of Variation in Dental Implant Dimensions (Length and Diameter) on Peri-implant Bone Stress/Strain Distribution: A Systematic Review

**DOI:** 10.12669/pjms.41.1.8991

**Published:** 2025-01

**Authors:** Arshad Jamal Sayed, Syed Fareed Mohsin, Kamlesh Kumar Garg, Muhammad Atif Saleem Agwan, Sabahat Ullah Khan Tareen, Mohammed Sulaiman Alruthea, Sasankoti Mohan

**Affiliations:** 1 Arshad Jamal Sayed Associate Professor, Department of Periodontology and Implant Dentistry College of Dentistry, Qassim University, Saudi Arabia. PhD Scholar, Pacific Academy of Higher Education, Research University, (PAHER) Udaipur, India, College of Dentistry, Qassim University, Saudi-Arabia; 2 Syed Fareed Mohsin Associate Professor of Oral Pathology, Department of Oral and Maxillofacial Diagnostic Sciences, College of Dentistry, Qassim University, Saudi-Arabia; 3 Kamlesh Kumar Garg Professor of Orthodontics, Pacific Dental College and Research Centre Udaipur, India; 4 Muhammad Atif Saleem Agwan Assistant Professor, Department of Conservative Dental Sciences, College of Dentistry, Qassim University, Saudi-Arabia; 5 Sabahat Ullah Khan Tareen Assistant Professor, Department of Conservative Dental Sciences, College of Dentistry, Qassim University, Saudi-Arabia; 6 Mohammed Sulaiman Alruthea, Associate Professor, Department of Prosthodontics, College of Dentistry, Qassim University, Saudi-Arabia; 7 Sasankoti Mohan Ravi Prakash, DMD, MDS, BDS Dentist and Independent Researcher, Hope Health Inc, 360 N Irby St. Florence, South Carolina, USA 29501

**Keywords:** Finite element analysis, Dental Implant, Stress/strain, Implant survival, Implant dimension, peri-implant bone loss

## Abstract

**Background & Objective:**

Currently, there are many implants in clinical use, making it hard to choose the right one for the patient. The success rate of an implant depends on its diameter, length, and direction of insertion in bone. In implant dentistry, Finite Element Analysis (FEA) simulates intraoral conditions in vitro and analyzes the effects of implant material, diameter, size, and other components related to oral structure on the implant and peri-implant tissues. The objective of the present systematic review was to evaluate the influence of variation in Dental Implant dimensions on Peri-implant Bone Stress/strain distribution, where Finite Element Analysis (FEA) is used as a method of analysis.

**Method:**

Research papers published in PubMed, EBSCO, Web of Science, MEDLINE, CINAHL database from January 2012 until December 2022 using the keywords, (finite element analysis) AND (dental implant) AND (length) AND (diameter). The modified CONSORT checklist was used to assess the quality of the included research.

**Result:**

Total hits from the original search were 402 (PubMed:127, Web of Science: 135 and CINHAL:22, EBSCO: 96, ProQuest:22). After duplicates were removed and titles and abstracts were screened, 371 articles were (n =371) selected for review. Of these, 342 were excluded and removed after initial screening, and 33 entire texts met the initial eligibility as per inclusion criteria. Four articles were further excluded in the final eligibility process and a total of 31 in vitro study articles were included for qualitative synthesis.

**Conclusion:**

The evidence from the most recent literature suggests that the use of FEA showed promising results in understanding the stress distribution surrounding the implant. It was discovered that the material of the dental implant and the prosthesis, the type of loading, the direction and magnitude of force (axial or non-axial), the quality and quantity of the bone that surrounds the implant, as well as other factors, all play a role in the maintenance of bone around dental implants.

## INTRODUCTION

Oral rehabilitation for patients who are either partially or completely edentulous can be achieved effectively using dental implants. Innovations in coating, material, and design technologies have made dental implants a viable alternative for nearly all clinical procedures. This has made it possible for implants utilized in almost all patients.^1^ Bone quality, bone density, and dimension are some of the most important considerations to consider when selecting the best implant.

The dimensions of the implant are highly influenced by the stress values and are dependent on the volume of bone that is readily available.^2^ Because there is such a wide variety of implants available today and because they are all currently being used in clinical settings; it can be challenging to choose the kind of implant that is going to serve an individual patient’s requirements in the most effective manner. Because there are no universally accepted selection criteria for implants, the decision to place them is almost always made based on the clinician’s familiarity with the patient’s bone size and their history of successful treatment.^3^

In addition, if an implant is subjected to occlusal force, there is a possibility that some mechanical issues will arise at the interface between the bone and the implant. Additionally, there has been a growing interest in the research of mechanical problems at the implant-bone interface. Therefore, it is essential to conduct both biological analysis and mechanical problem-solving to ensure a good outcome with implant therapy.^4^ The stress over the bone surrounding an implant can affect the implant’s success rate, and this stress can be affected by several factors such as the implant’s diameter, length, and the direction of insertion into bone.^5^

The results of the numerous ongoing in vivo and in vitro studies that have attempted to evaluate the impact of implant dimensions on the stress that is transmitted to the peri-implant tissue have not yet provided a definitive answer to the complex question being asked.^6^,^7^ Too little study has been conducted to determine how bone geometry affects implant-related stress at crestal bone. This is a problem because the dimensions of the implant, the stress applied to it, and the geometry of the bone are all interconnected and should not be studied in isolation.^8^ Both the total stress and how it is distributed affect how long an implant lasts. Possible bone resorption due to high-stress concentration at the peri-implant bone due to excessive implant loading.^1^,^9^

Because of this, effective examination methods using radiograph, and computed tomography (CT) devices for three-dimensional visualization have evolved, allowing for precise diagnoses.^10^ Using these measurements, it has been claimed, 3D FE models of bones can be made that accurately capture the bone’s subtle morphology. The use of finite element analysis (FEA) for biomechanical analyses is relatively new to the field of biological research.^11^ Dental research often uses finite element analysis (FEA) to determine how implant design factors like mini-screw diameter and length, thread shape, thread size, degree of taper, the influence of taper length on insertion torque, pullout strength, and stiffness affect stress distribution in the bone supporting the implants.^12^,^13^ FEA Models are used to evaluate the materialistic properties of anatomies.^14^

These models replicate the living structures, and analysis of stress is performed by applying mechanical forces to the models.^6^,^15^ Digital images, such as CT scans and MRI scans, are used to extract information about the geometry and properties of bones, which is then input into an FEA model with the assistance of a number of different software.^6^ The FEA model can display a variety of stress patterns, such as the Von Mises stress and the principal stress.^16^ The Von Mises stress is used to determine the yield strength or fatigue strength of ductile materials, while the principal stress is directly measurable and less theoretical.^17^,^18^

The stress analysis performed using the FE method exhibited information on the distribution of stress for any form of implant.^12^ FEA analysis provides a significant benefit to dentistry by in vitro studies and analyzing the effect of varying the number of implants, the material, and the effect of other components on anatomical structure.^11^ Evidence of the technique’s actual clinical efficacy and accuracy is provided through systematic reviews. Considering this, the present systematic review was conducted with the intention to understand the influence of Finite Element Analysis (FEA) on the variation in Dental Implant on Peri-implant Bone Stress/strain distribution by synthesizing the data from several research.

## METHOD

This review was registered in Prospero (CRD42022322045 dated April 30, 2022) and conducted in accordance with the Preferred Reporting Items for Systematic Reviews and Meta-Analyses (PRISMA) guideline (International Prospective Register of Systematic Reviews). No institutional review board permission was required because of the nature of the current investigation.

### Focused PICO question:

The PICO question was developed to identify the appropriate studies to answer: “Using Finite Element Analysis (FEA), does variation in dental implant dimensions Influence peri-implant bone Stress/strain distribution? Where (P) denotes participants (cases requiring replacement of missing teeth), (I) indicates intervention (Finite Element Analysis, stress/strain distribution), (C) represents the comparison (manual variation in implant dimensions, variation length of an implant (short, regular or longer), and diameter of an implant (regular, wide, ultra-wide), and (O) represents the outcome (distribution of stress/strain in periapical bone, survival rate of the implant).

### Search strategy:

All the papers outlining research questions were searched for in PubMed, EBSCO, Web of Science, MEDLINE, CINAHL database from January 2012 until December 2022. All the relevant papers were found using the search method using the keywords, (finite element analysis) AND (dental implant) AND (length) AND (diameter). Additionally, all relevant articles’ reference lists were hand-searched as well. To find additional related studies, a manual search was carried out on the hosting publishers (Wiley, Science Direct, and Springer) as well as separately on the renowned implant journals.

### Eligibility criteria:

For studies to be considered for inclusion in the systematic review, they must satisfy the following inclusion criteria.: Articles published in the English language up to December 2022, experimental studies evaluating stress/strain around implant using finite element analysis. The exclusion criteria encompassed: studies evaluating the finite element analysis effects of variation in dimension with distribution of forces, clinical studies, histological studies, case reports, Review of literature, systematic reviews, editorial papers, magazine articles.

### Study selection:

The research title, abstract, and keywords of the pertinent publications were independently reviewed by three investigators (AJS, KKG and SFM) to determine their eligibility. Then, all possibly eligible paper’s full texts were retrieved and carefully reviewed to find research that matched all inclusion requirements. A list of the articles to be included in this evaluation was established after any disagreements were discussed with the third reviewer (MSA).

### Data extraction:

Titles and abstracts of the chosen studies were independently evaluated by three authors (AJS, SFM, and KKG) who screened the titles and selected the abstracts for full-text inclusion. Using the Mesh terms, following the inclusion and exclusion criteria all relevant full-text articles were retrieved. In case of any disagreement between the two review authors, it was resolved with the arbitration of two senior authors (MASA and MSA). The data extracted will include Study ID, Author Year of publication, Study Design, details of the intervention and comparison conditions, study methodology, result and times of measurement, Implant stability value, outcomes, and statistics. Data Extraction was carried out by four authors (AJS, SFM, SKT, and MSA).

### Risk of bias:

Experimental studies using FEA analysis where the distribution of forces around implants with varying dimensions are included. Hence, wherever possible we will assess the following domains:

- Random sequence generation (selection bias);
- Blinding of participants and personnel (performance bias);
- Incomplete outcome data (attrition bias);
- Selective outcome reporting (reporting bias); This provided the rationale for our judgment of that domain as at low, high or unclear risk of bias which was done by the two main authors (AJS and SFM) independently, and the third and fourth author (MASA and MSA) was consulted when there was discrepancy in judgment.

### Data analysis:

Data extraction revealed significant heterogeneity among the included papers, preventing the execution of a meta-analysis. Instead, information was gathered into a table and used to create a descriptive summary that detailed the study’s characteristics and results.

### Quality assessment:

The modified CONSORT checklist was used for in vitro and in vivo investigations to assess the quality of the included research. Following the application of the checklist, the average compliance of all the articles as well as the minimum and maximum were noted. Each parameter’s compliance percentage was also determined.

## RESULTS

### Search results:

Total hits from the original search were 402 (PubMed:127, Web of Science: 135 and CINHAL:22, EBSCO: 96, ProQuest:22). After duplicates were removed and titles and abstracts were screened, 371 articles (n =371) were selected for review. Of these, 342 were removed after initial screening, and 35 entire texts met the initial eligibility as per inclusion criteria. Four articles were further excluded in the final eligibility process and 31 in vitro study articles were included for qualitative synthesis.

### Characteristics of included studies:

The characteristics of all included studies are summarized in Table-I.. All the included studies.^1^,^3^,^5^,^7^,^11^,^19^-^44^ were in vitro studies, and are published up to December 2023. The included studies all had a common goal connected to the current research question; however, each study carried out its tasks in a unique manner and used a diverse set of parameters to analyze the stress and displacement of the implant. Only six of the 31 studies didn’t consider the mandibular posterior segment for their FEA analysis, but 15 of the studies did.^5^,^20^,^30^,^34^,^37^,^38^,^41^ While study done by Chatzigianni A et al.^23^ used bovine rib for FEA analysis. In addition, most of them performed the analysis on either the cortical or the cancellous layer or both. However, the quality analysis revealed that there was some variation in the bone. Osseo integrated bone was utilized in most studies, while Niroomand MR et al.^40^ used B/2 bone; Bone type D2 by Robau-Porrua A et al.^3^, Type-4 by Demenko V et al.^38^ and Type-III bone by Lu YJ et al.^34^ To carry out FEA analysis, a variety of specifications were taken into consideration, and these can be compared across the various studies that were included. It has been found that the Solid Work 3D programming language was utilized by the overwhelming majority of the studies.^20^,^24^,^31^,^32^,^36^,^44^ followed by Pro/ ENGINEER Wildfire.^21^,^22^,^31^,^35^

**Table-I:** Data Extraction of Included Studies

	Material & Methods: FEA Analysis Specifications	Material & Methods: Type of implant selected; Amount of load applied for FEA	Results of FEA Analysis	Summary
Authors & Year of Publication	1.Teeth or Area considered for FEA analysis with Bone layers considered (Cortical Bone - CBor Cancellous / Trabecular Bone - CT) 2. 3D program Software & FEA Software.	Number / Type/ Design of implants (I) placed, Length (L) and Diameter (D), Implant abutment connection (IAb) / Implant supra-structure (ISt) construction. Load applied: (Axial (AxL), Inclined / Oblique Load (ObL) / Lateral load (LaL))	Stress/strain (Von Mises Value- VMS), Maximum Displacement (µ) in Cortical and Cancellous Bone (CB & CT)
Fellippo Ramos Verri et al 16 (2007)	1. Mandibular RPD models with distal extension. 2. AutoCAD 2000 & ANSYS 5.4	I-Nobel Biocare, L-7mm and 13 mm, D-3.75-mm (standard), and 5.00-mm implants (MKIII). AxL: 50 N on each cuspid point, divided into 5 points of 10 N, 100 N in model A and 400 N in the other models.	RPD implants showed reduced tension around implants; In spongy bone, stress decreased with increasing diameter and length.	Larger implants improve stress distribution and reduce displacement.
Heng-Li Huang et al 17 (2008)	1.Maxillary Human Skull model – premolar-second molar with CB + CT2.SolidWorks & ANSYS,	I- Six (cylindrical, threaded, stepped, step-thread, long-length of threaded, and wide-diameter of threaded). L-Std. (S) - 11.5 mm, long (L)- 13.5 mm, D- Std. (S) - 4 mm, Wide (W)- 5 mm. ObL-129 N 45° applied lingual cusp of the crown.	Threaded implants lowered bone stress by 30% compared to cylindrical and stepped implants; longer implants reduced stress by 13–26%.	Immediate loaded threaded implants enhanced stability; longer and wider designs minimized stress.
Liang Kong et al 18 (2009)	1. Posterior mandible segment with CB & CT.Thick layer of CBsurrounding dense cancellous bone (type B/2 bone), CBthickness - 1.3 mm. 2. Pro/ENGINEER Wildfire & ANSYS.	I-9 models Straumann, L- 6-16mm, D- 3-5mm, ISt- Full-porcelain superstructure (mandibular first molar) was achieved using a 3D sensing system. AxL- Forces of 100N and 30 N, LaL-Forces of 100N and 30 N applied Buccolingually at a 45-degree angle on the buccal cusp.	Max EQV stress in CB (73.3MPa) and cancellous bone (74.9MPa); increasing dimensions minimized stress. Max displacement in the implant-abutment 64.6 (axial).	Optimal dimensions minimized stress and displacement in type B/2 bone immediate-loading implants.
M. Mohammed Ibrahim et al. 19 (2010)	1.Non-Specific. CBanalysis. 2. Pro-e wildfire 4.0 software & ANSYS software through IGES.	I-Six models Zimmer. Group 1: Screw-vent tapered. Group2: parallel, L- 13mm. D- 7, 4.1 & 4.7mm, AxL- 114.6 N, ObL- 17.1N Lingual LaL- 23.4N disto-mesial.	Increase in implant D in Group I and Group II from 3.7 to 4.1 mm and from 4.1 to 4.7 mm with constant 13 mm length for screw-vent tapered; parallel designs distributed stress more evenly.	Diameter and design significantly impact bone stress distribution as tapered showed higher stress than the parallel design.
Hsuan-Yu Chou et al 7,20 Ivan Onone (2010)	1. Mandible premolar area with CB & CT. 5 different insertion depths and 2 different levels of alveolar bone quality. 2. Pro/ENGINEER Wildfire 2 & ANSYS 11.	I- Bicon, L- 5.99 & 10.96mm. D- 3.5 & 5.0 mm, ObL: 100 N on the abutment at 11 degrees to implant axis in the Bucco-Lingual plane.	Wide Diameter Short (WDS) and narrow diameter long (NDL) implant types, bone volume experiencing strain levels less than 200 με, and/or greater than 3000 με, Strain levels were lower in grooves than at thread peaks.	The peri implant bone strain was higher in WDS vs. NDL implant. Improved stress distribution was noted with thread patterns.
Athina Chatzigianni et al. 21 (2011)	1. Bovine rib bone specimen with CB & CTand Mini Implant. 2. Micro-CT & MSC.Marc/Mentat 2007 R1.	I- LOMAS mini-implants L&D-(1.5 × 7 mm, 1.5 × 9 mm, and 2 × 7 mm). LaL: Mesio-distal forces of 0.5 N	At bone margin, CB had 4.4 MPa. The CT’s total maximum strains are 1195(μ). localized strains at thread peaks; minimal displacement and rotation observed.	Both Numerical (FEA) and Experimental study results were similar with respect to implant displacement and rotation.
Zeev Ormianer et al.^22^ (2012)	1. Non-Specific CB & CT, and dental implants. 3 cylindrical Bone models, 20mm x 2mm thickness with Peri-implant bone thickness of 1- 5mm. 2. SolidWorks Pro 2006 & ANSYS 11.0.	I-One piece Zimmer (1P) or Two Piece (Tapered Screw Vent, Zimmer- 2P), D-1P implant & 2P with variable diameters (3.0, 3.7, 4.1, 4.7, or 6.0 mm), AxL: A 222 N of occlusal force applied at a 30-degree angle with a 1.5 mm buccolingual vertical axis of the implant.	Stress levels in 3.0 mm 1P implants in low-density bone may influence marginal bone stability. Low peri-implant bone thickness increased bone stress.	Implant diameter and peri-implant bone thickness affected stress distribution in bone,1P implants should be limited to dense bone.
Marcelo Bighetti Toniollo et al. 23 (2012)	1.Posterior left side segment of the mandible with CB & CT. 2. Ansys-Workbench 10.0 (TX).	I- Morse Taper, L- 5, 11, 13mm, D- 2.54mm, 2.96mm, AxL: 365N & 200N, ObL: 365N & 200N & LaL: 365N & 200N.	Short implants (5mm) had 50% higher stress on cortical bone compared to longer implants (11mm and 13mm).	Short implants may require occlusal adjustments to prevent overload.
Bobin Saluja et al. 24 (2012)	1. Mandible with Nonspecific bone layers. 2.3D-VISI soft & Abacus software (FEA)	I- INCIDENT system (External Hex, Internal Hex and Ball, Socket type), L- 8, 10 and 12 mm. D- 3.5, 3.8, 4.2 and 5 mm, AxL- 100N	Length did not significantly affect stress distribution; increased diameter enhanced contact area.	Wider implants enhance stability and reduce stress.
Mehmet Bayraktar et al. 25 (2012)	1. Edentulous mandible (2^nd^ premolar and 1^st^ molar) with 10- & 5-mm mandibular height and width respectively. 2. CAD (ILUMA) & Autodesk, Inc, CA).	I- Straumann, L- (6, 8, and 10 mm), D- (3.5, 4, and 5 mm). Implant C/I ratio: (1, 1.5, & 2). IAb:A 2-unit fixed bridge, ISt- metal thickness - 0.8 mm, porcelain thickness - 2.0 mm. ObL: A 100 N 30° force.	Increased stress on short implants; wider diameters provided better support; 2:1 C/I ratio models had the highest stress values.	Crown height affects peri-implant bone stress. Wide-diameter posterior implants can support high - C/I two-unit fixed restorations.
Toniollo MB et al. 26 (2013)	1. Posterior left side segment of the mandible with CB & CT. 2. Ansys Workbench 10.0.	L- 5mm, 11mm, 13 mm, D- 4 mm, IAb- heights 3.5 mm for 13 mm and 11 mm (regular) and 0.8 mm for 5 mm (short). ISt- ceramic crown 2 to 3 mm thickness. ObL-365 N for molars and 200 N for premolars.	Shorter abutments (0.8 mm) resulted in lower Von Mises stress than longer ones (3.5 mm); larger crowns increased surface stress.	Shorter implants with larger crowns increased implant surface stress.
Istabrak Hasan et al. 27 (2014)	1. Bone sample width and length of 15 mm * 25 mm with CB + CT. Bone Quality: CBthicknesses 2-3 mm, fully Osseo integrated. 2. MSC Marc/Mentat 2010 (USA).	I- Nine self-tapping (Germany), L- S - 9-13 mm, M- 7-13 mm, L - 13 mm. D- S - 3.3 mm, M- 3.7-4.2 mm, L - 4.8-5.5 mm, AxL- 300 N, ObL- 300 N 45° from the implant’s long axis.	Maximum stresses decreased with larger diameters (3.7 - 5.5 mm). Lateral loading caused an increase of the VMS with a thin cortical layer of 2 mm. For AxL 0.012 mm displacement with 2 mm cortical thickness, 0.007 mm µwith 3 mm cortical thickness.	Self-tapping implants under axial or later loading provide an acceptable loading of the bone that was within the physiological range.
Rudi C et al 28 (2014)	1. Maxilla surrounding the first molar in the maxillary right quadrant with CB & CT. 50% osseointegration “soft quality” or type 4 bone. 2. Strand7 (Strand7Pty) FEA system.	I- Bicon, Neodent, Nobel BioCare, and Straumann, L- 6 - 8.8 mm. D- 3.75, 4, 4.1, 4.5 mm, ISt- Height: 12mm. ObL- 200 or 1,000 N on four crown cusps 45-degree inclination.	Straumann had highest cortical bone stress at 200N (27.70) and Neodent the lowest (23.59). At 1000N, Straumann implant had 138.48 stress and Neodent 117.97.	Type 4 bone is best treated with Nobel BioCare and Biocon systems. Straumann implants experience the highest stress at 200 and 1000 FM, while Biocon has the highest stress among all implant systems.
Eda OZYILMAZ et al 29 (2014)	1.Mandible with CB & CTlayers. 2.SolidWorks 2013 & Ansys Workbench.	I- Twelve, 3 Models, L- 5-7mm, D- 5mm, IAb- 3.5, 4, 5, & 5mm, AxL- 40 -100N	VMS values decrease with abutment length in all models, from 8.03 MPa in 3.5mm and 5mm implants to 5.14 MPa in 3.5mm and 7mm implants.	Increasing implant length and decreasing abutment length reduce stress values on dental implants and abutments.
V. Demenkoa, I et al. 30 (2014)	1.Human mandible with CB & CTlayers. 3.SolidWorks 2008, & CosmosWorks 2005.	I- TPS Cylinder-shaped non-threaded, L- 0–17.0mm, D- 5 – 7.0 mm, AxL- 114.6N, LaL- 23.4N in lingual and distomesial.	2.5 mm and 3.0 mm implants had the highest VMS (75 MPa). 7.0 mm diameter and 17.0 mm length implants had the lowest VMS (9.8 MPa).	Implant length and diameter reduce cortical bone stress. Threaded implants are more reliable.
K Raghavendra R et al. 31 (2014)	1.Mandible lower first molar region with CB & CT. 2.CATIA modeling & ANSYS 12.0 (USA).	I- 8 models with crown, L- 10 & 14 mm, D- 4 & 5 mm, ISt- Porcelain, AxL- Group I 250 N & 300 N, Group 2- 400 N, 700 N, ObL- Group I (100 N, 200 N), Group 2 (300 N, 400N).	Increasing implant diameter, cortical bone stress levels decreased in threaded implants (75.5Mpa) under 400N force and increased in non-threaded implants (92.5 MPa). Variation in length was non-significant on stress distribution.	Wider diameter threaded implant with a shorter length has a favorable distribution of stress and strain.
Ying-juan Lu et al 32 (2015)	1. Maxilla (2nd molar) with CB & CTwith CBthickness of 1.5–2.0 mm. 2. Mimics 10.0 software & ANSYS 13.0.	I-Tomas micro-impl(Germany), L- 6 mm, 8 mm, 10 mm, and 12 mm, D- 1.2 mm, 1.6 mm, and 2.0 mm. AxL- Single Force(SF) of 200 g loaded near the middle, Composite force (CF) of 200 g and 6 N. mm torque in the cross groove.	The Max EQS of the cortical bone around mini-implant in Group CF was higher (P<0.05) than that in Group SF. SF & CF nonsignificant (P>0.05) bone stress when diameter of mini implant was 1.6 mm or 2.0 mm.	Diameter of mini-implant is better to be larger than 1.2 mm when a mini-implant is used in a torque control of tooth.
Helen Mary Abraham et al.^33^ (2016)	1. Nonspecific bone with CTlayers. 2. Pro Engineer Wildfire (USA) & ANSYS.	I- 13mm, IAb- 6.5 mm, Ni-Cr restoration L- 8mm, D- Regula platform (4.3 mm), Narrow platform (3.5 mm), Ni-Cr (8mm). ISt- 8 mm height and 8 mm diameter with 1.5 mm thickness. ObL- (35.6°) load of 90 N.	Regular platform (RP) implants reduced stress by 3.38% compared to narrow platforms (NP).	Regular platform implants reduce peri-implant stress in healthy as well compromised bone conditions.
R. Eazhil, Siva Vadivel S et al. 34 (2016)	1.mandibular molar segment with CB. 2.CATIA software USA & ANSYS v 10.0.	I- Nobel BioCare Tapered threaded, L- 13.0–16.0 mm, D- 5–5.0 mm. Axl- 14.6N, ObL- 118.2N at 75-degree angle, LaL- lingual 17.1N, distomesial 23.4N.	The maximum stress was concentrated around the neck of the implant on the mesio-lingual region of the socket.	Decrease in von Mises stress as the implant diameter increased.
K. Jomjunyong et^35^ (2017)	1.Posterior Maxilla with CB & CTlayers of Type 3 density. 2.Solidworks 2006 & ANSYS 5.7.	I-(PW Plus®) Tapered, L- 10 mm, D- 5 mm. Connected with fixed partial dentures, Full metal crown & Gold crown. AxL- 200N, ObL- 200N 30 degrees.	VMS of six models in cortical bone ranges from 40.43 - 42.39 (MPa), and in cancellous bone is 4.89 - 8.03(MPa).	Prosthetic design and Implant length impacts stress distribution significantly.
Brunilda Gashi C et al 5 (2019)	1. Atrophic posterior maxilla with CB(1.5mm) and CTlayers, 4 models with 2 or 3 osseo-integrated implants and fixed partial dentures 2. Rhinoceros 4.0, (USA).	I- Astra Tech, Sweden), 4-mm long 20◦ angled abutments, 2 each implant in 4 models. L- 8 mm for 2nd PM & M or 13 mm for 1st PM, D- 4 mm. M1, M2, M3 & M4 Implant supported prosthesis. AxL- 2 points, 100 N on PM, & at 4 points, 140 N on M. ObL- 2 points, 200 N on PM & at 4 points, 280 N on M. LaL- 2 points, 30 N on PM & at 4 points, 45 N on M.	The M1 model had the highest stress; bicortical implants reduced stress significantly. M1 showed maximum displacement (0.00724mm) followed by M2 (0.00324mm), M3 (0.00302mm) and M4 (0.00182mm).	Cantilever implant prosthesis (M1) may induce high bone stress, while connected crowns supported by three implants (M3), may induce low bone stress.
Vladislav Demenkoa et al 36 (2019)	1. Posterior maxilla with Type IV bone with CB & CTlayers & implants. 2. ANSYS 15(USA) software.	I- 6 Short implants, L- range of 3.3, 4.1, 4.8, 5.4 mm. D- 4.5, 5.5, 6.5, 7.5, 8.5 mm, placed bi-cortically in the maxillary first molar area. AxL:114.6 N, ObL- 118.2 N & LaL- 17.1 n in lingual direction, 23.4 N in mesio-distal direction.	The largest implant (5.4 × 8.5 mm) had a 2.5-fold higher ultimate functional load (UFL) increase than the lowest (3.3 × 4.5 mm). UFL dropped from 83% for a 4.5 mm implant to 73% for an 8.5 mm implant when implant D increased from 3.3 to 5.4 mm.	Wider and shorter implants minimize stress, especially in type IV bone.
Gowthama Raaj et al 37 (2019)	1.Edentulous mandible. Five posterior bone models’ segments with CB & CT. 2. Creo 2.0. software & ANSYS Work 17.0.	I- Nobel Replace Tapered, L- 10mm & 11.5mm, D- 3.5 & 4.3mm, AxL- 100N force, ObL- 50N force & LaL- 50N force	Under axial and non-axial loads, 3.5 × 10 mm implant demonstrated maximum VMS in both CB & CT bone, while 4.3 × 11.5 mm implant showed the least.	Cortical bone stress is lowest with larger implants. Implant length minimizes cancellous bone stress. Diameter and length critically affect stress values.
Amanda Robau Porrua et al 3 (2020)	1. Mandible segment corresponding to the premolar region with CB & CTand dental implants. Bone- D2, 2mm CBthickness. 2. Free CAD 0.16 & Abaqus/CAE (France).	I- 12 Implants with variation in L- 10-13 mm & D- 3.8-4.5 mm, and porosity (Dense, 40% & Medium 17% porous). AxL- 17.1 N, 114.6 N, and 23.4 N, equivalent to 118.2 N forces. ObL- 17.1 N, 114.6 N, and 23.4 N, (75°) equivalent to 118.2 N. LaL- 17.1 N, 114.6 N, and 23.4 buc-ling & Mesial, equivalent to 118.2 N.	Highest VMS values (0.7–0.9 MPa) in the mesial site along the implant neck in peri-implant CB. Buccal and lingual sites had the highest VMS (Strain) values interface axially.	In all simulations, the coronal area of the implants (<140 MPa) had the maximum stress. Diameter and length significantly influence stress distribution.
Mohammad Reza N et al.^38^ (2020)	1. Mandibular 1st Molar region with CB(1.3-2mm) and CTlayers of Type of B/2 bone, ITI and titanium threaded implant. 2. ANSYS Workbench 19.1.	I- 45 models with Thread Depth (0.25, 0.35, 0.45 mm), Thread Width (0.1, 0.2, 0.3 mm), Thread Pitch (0.75, 1.05, 1.35mm) and Thread Angle (25°, 30°, 35°). L- 10mm, 13mm, 16mm, D- 3.4mm, 3.8mm, 4.1 mm. ISt- ceramic crown with 2 mm thickness. ObL- 100 N buccolingual force with a 45° to the center of crown.	The VMS in implant and bones demonstrates that crestal cortical bone receives and dissipates more VMS. Length increases implant-cancellous bone contact, reducing displacement. TW and TA reduce implant displacement.	Optimized thread design reduces stress, improves stability, and reduce bone resorption.
Doriana A F et al^1^ (2020)	1. Mandibular model with 2mm thick CBand an internal CT. Class II bone (atrophic) with (bone height >10 mm, bone crest 2.5–5 mm). 2. Autodesk Fusion360 & Autodesk Mechanical version 2017 (USA).	I- 2 Classic tapered threaded. L- 5.0 mm - 13.0 mm. D- 3.3 mm to 6.0 mm. ISt- conic 10 mm length abutment. AxL- 114.6 N, LaL- 17.1 N lingual direction, and 23.4 N distomesial direction.	The 3.3D/5L implant increased CB & CT bone stress in resorbed mandibles. Bone stress was lowest in 3.7D/8L implant. In bone augmented ridge, cortical bone stress was maximum with 3.7D/13L and lowest with 5D/11.5L.	Fully augmented bone allows clinicians to utilize larger implants, reducing peri-implant stress. Diameter and length reduce stress equally.
Akikazu Shinya et al. 11 (2021)	1.mandibular first molar with CB & CTlayers. 2.Mechanical Finder ver. 5 (Japan) & Precision workstation 670, Dell Inc.	I- 12 mandibular 3D FE models. L- 9,11,13, & 16 mm. D- 3.8, 4.3-, & 6.0-mm. ObL- 50 N & LaL- 50 N.	All implants had high stress at the neck bucco-lingually. With implant diameters of 3.8, 4.3, and 6.0 mm, stress values were 6.0, 4.5, and 3.2 MPa, respectively.	The stress on the peri-implant bone was found to decrease with increasing length diameter of implant.
Erfan Sheikhan et al.^39^ (2022)	1.mandibular bone block with 20-mm height and 12-mm buccolingual and mesiodistal width with CB & CT. 2. The CATIA V5 program & e-ANSYS 18.2.	I- 5 Models geometric variables, L- 8, 10, 12 (mm). D- 3.5, 4.5, 5.5 (mm), Taper: 0, 2, 5(°), Thread depth: 0.2, 0.5 (mm). Thread Angle: 0, 15, 25 (°). AxL- 100 N, LaL- 20 N.	Increasing diameter 3.5 to 5.5 mm and thread depth reduced strain by 35%; thread angle had the least impact.	Thread depth and diameter had the greatest effect on reducing compressive & tensile bone strain.
Hyeonjong Lee et al. 40 (2022)	1.Bone density (low, high) with CB & CT. 2.3-matic Research 9.0 & ABAQUS 6.14.	I- Twelve 3D Models (Osstem I). L- 10 (mm). D- 3.5 mm, 4.0 mm, and 4.5 mm). IAbtype: Internal Bone level (IB) & Internal Tissue level (IT). AxL- 6-point contact 200 N, ObL- 3-point contact 100 N.	Maximum & minimum principal strain in low density model: Cortical bone 3103.6 MPa, Cancellous bone 4,630.3 MPa. Minimum principal strain (high density): Cortical bone -7,253.2 MPa, Cancellous bone -4,133.2 MPa. VMS (MPa): Implant - 364.3, Abutment - 402.7.	Implant connection type influenced stress more than implant diameter. Tissue-level connections were more effective for stress distribution.
Ivan Onone Gialain et al.^41^ (2022)	1. Anterior maxilla, Maxillary lateral incisor with CB & CT. D3 Bone. 2. Rhino3D software (version 7, WA) & MSC.Apex and MSC.Marc programs (USA).	I- Eight models (Neo-Poros, Brazil). L- 10 and 13mm, D- 3.25, 3.50, 3.75-, & 4.00-mm. IAb- The cone-morse abutment (NeoDent, Brazil), Crown: full-ceramic. Obl- 50 N, 100 N.	The only trabecular bone model with a low peri-implant bone resorption risk index was 4.00mm x 13mm. Smaller diameters (3.5 mm) increased resorption risk.	Diameter choice is crucial for bone preservation. Smaller diameters (3.5 mm) increases resorption risk.
Eduardo Anitua et al. 42 (2022)	1. Non-specific bone with CB & CTlayers. 2. Solidworks Simulation Premium 2020.	I- BTI core. L- 16.5mm & 16.5mm. D- 15.5mm & 20mm. AxL- 200N. ObL- 200N 17-degree, 30-degree, 45 degrees.	At 45°, IMI had the highest VMS and in Bone type I, II, and III increased by 69.4, 83.8, and 138.5 MPa. In delayed loading, extra-short implants in axial position raised stress by 4.8–6.5 MPa in Type III bone compared to standard length.	Axially positioned short implants perform better under stress for single-unit restorations in an atrophic mandible.

Similarly, ANSYS Workbench software used by majority of studies followed by MSC Software by^23^,^29^,^43^, and only one study^1^ used the FEA program for analysis. In addition, tetrahedral elements with ten nodes were the most common type of element, whereas only a select few authors^3^,^5^,^32^,^34^ utilized mesh for their analyses. However, there was no specific range for elements used in the included studies. Learning about the properties of the implant material and the analysis of its coefficient of friction was very interesting. Titanium was used as the implant material in most studies, and the coefficient of friction of titanium in cortical and cancellous bone was measured. When determining the stress and deflection properties of materials, Young’s Modulus and Poisson’s ratio were two of the most useful tools. Readings for this measure were not able to be compared across studies because they differed from one another.

Additionally, the type of implant that was utilized for FEA was contrasted with implants and their diameters. There were four types of implants commonly used in the studies, threaded^1^,^17^,^38^ tapered^36^,^37^,^39^ Stranumann design^21^,^27^,^30^ and Screw vent tapered design.^22^,^24^ The implant length and diameter were ranged from [6 to 17mm; 2.54 to 6mm] respectively. When comparing the types of loads among the included studies, five studies^3^,^5^,^36^,^38^,^39^ compared all three types of loads (axial, oblique, and lateral) to better understand FEA analysis.

Many studies used 100 to 200N of load for axial and oblique stress while Lu YJ et.al.^34^ used 50N stress for lateral load. When the stress analysis was compared, the maximum EQV stress in cortical bone was observed with an implant that had a diameter of 3.5 mm and a length of 10 mm. This corresponded to a value of 73.3 (axial) and 81.0 (Buccal) in MPa.^21^,^27^,^32^ Bone stress levels increased as peri-implant bone thickness decreased for all implant designs and diameters. It was also found that the maximum value of Von Mises stress in the bone around the implant was reduced when the implant had a screw-vent tapered or parallel design.^22^,^24^,^28^

### Quality of included studies:

The detailed results of modified CONSORT evidence quality evaluations are shown in Table-II. Overall mean compliance of case reports was 71% with a maximum score of 100% (Hasan I et al),^29^ 90% (Huang HL et al.)^20^ and a minimum score of 50% (Ozyilmaz E et al.)^31^ IT was also observed that, majority of studies did not mention Statistical methods used to compare groups for primary and secondary outcomes, source of funding and where the full trial protocol can be accessed.

**Table-II:** CONSORT Check List Analysis for Experimental FEA studies.

	Author Name & Year of Publication	Abstract	Introduction		Materials and Method								Results	Discussion	Funding	Protocol	%

		A	B	C	D	E	F	G	H	I	J	K	L	M	N	O	
1	Fellippo Ramos Verri et al^16^ (2007)	Y	Y	Y	Y	Y	Y	NA	NA	NA	NA	N	Y	Y	N	N	70%
2	Heng-Li Huang A et al^17^ (2008).	Y	Y	Y	Y	Y	Y	N/A	NA	N/A	N/A	Y	Y	Y	Y	N	90%
3	Liang Kong et al^18^ (2009).	Y	N	Y	Y	Y	Y	Y	NA	NA	NA	N	Y	Y	N	N	60%
4	M. Mohammed Ibrahim et al^19^ (2010).	Y	Y	Y	Y	Y	Y	NA	NA	NA	NA	N	Y	N	N	N	60%
5	Hsuan-Yu Chou et al 7,20 (2010).	Y	Y	Y	Y	Y	Y	NA	NA	NA	NA	N	Y	Y	N	N	70%
6	Athina Chatzigianni et al^21^ (2011).	Y	Y	Y	Y	Y	Y	Y	NA	NA	NA	Y	Y	Y	N	N	85%
7	Zeev Ormianer et al^22^ (2012)	Y	Y	Y	Y	Y	Y	NA	NA	NA	NA	N	Y	Y	N	N	70%
8	Marcelo Bighetti Toniollo et al^23^ (2012).	N	Y	Y	Y	Y	Y	NA	NA	NA	NA	N	Y	N	Y	N	60%
9	Bobin Saluja et al 24 (2012).	Y	Y	Y	Y	Y	Y	NA	NA	NA	NA	N	Y	Y	N	N	70%
10	Mehmet Bayraktar et al^25^ (2013).	Y	Y	Y	Y	Y	Y	NA	NA	NA	NA	N	Y	Y	Y	Y	90%
11	Marcelo Bighetti Toniollo et al^26^ (2013).	Y	Y	Y	Y	Y	Y	NA	NA	NA	NA	N	Y	Y	N	N	70%
12	Istabrak Hasan et al^27^ (2014).	Y	Y	Y	Y	Y	NA	NA	NA	NA	NA	Y	Y	Y	Y	NA	100%
13	Rudi C et al 28 (2014).	Y	Y	Y	Y	Y	Y	NA	NA	NA	NA	N	Y	Y	N	N	70%
14	Eda OZYILMAZ et al^29^ (2014).	N	Y	Y	Y	Y	Y	NA	NA	NA	NA	N	Y	N	N	N	50%
15	Demenkoa V et al^30^ (2014).	N	Y	Y	Y	Y	Y	NA	NA	NA	NA	N	Y	Y	N	N	60%
16	K Raghavendra Reddy 31 (2014).	Y	Y	Y	N	Y	Y	NA	NA	NA	NA	N	Y	Y	N	N	60%
17	Ying-juan Lu et al^32^ (2015).	Y	Y	Y	Y	Y	Y	NA	NA	NA	NA	Y	Y	Y	N	N	80%
18	Helen Mary Abraham et al^33^. (2016).	Y	N	Y	Y	Y	Y	NA	NA	NA	NA	N	Y	Y	Y	N	70%
19	R. Eazhil ET AL^34^ (2016).	Y	Y	Y	Y	Y	Y	NA	NA	NA	NA	N	Y	Y	Y	N	80%
20	K. Jomjunyong et al^35^ (2017).	N	Y	Y	Y	Y	Y	NA	NA	NA	NA	N	Y	N	Y	N	60%
21	Brunilda Gashi C et al^5^ (2019).	N	Y	Y	Y	Y	Y	NA	NA	NA	NA	NA	Y	Y	Y	N	80%
22	Vladislav Demenkoa et al^36^ (2019).	y	y	y	y	y	y	NA	NA	NA	NA	N	Y	Y	Y	N	80%
23	Gowthama Raaj et al 37 (2019).	Y	Y	Y	Y	N	Y	NA	NA	NA	NA	N	Y	Y	Y	N	70%
24	Amanda Robau Porrua et al^3^ (2020).	N	Y	Y	Y	Y	Y	NA	NA	NA	NA	N	Y	Y	N	N	60%
25	Mohammad Reza N et al^38^ (2020).	N	Y	Y	Y	Y	Y	NA	NA	NA	NA	N	Y	Y	N	N	60%
26	Doriana A F et al^1^ (2020).	Y	Y	Y	Y	Y	Y	NA	NA	NA	NA	N	Y	Y	Y	N	80%
27	Akikazu Shinya Et al^11^ (2021).	N	Y	Y	Y	Y	Y	Y	NA	NA	NA	N	Y	Y	Y	N	70%
28	Erfan Sheikhan et al. 39 (2022).	Y	Y	Y	Y	Y	Y	NA	NA	NA	NA	N	Y	Y	Y	N	80%
29	Hyeonjong Lee et al. 40 (2022).	Y	Y	Y	Y	Y	Y	NA	NA	NA	NA	Y	Y	Y	N	N	80%
30	Ivan Onone Gialain et al. 41 (2022).	Y	Y	Y	Y	Y	Y	NA	NA	NA	NA	N	Y	Y	Y	N	80%
31	Eduardo Anitua et al. 42 (2022).	N	Y	Y	Y	Y	Y	NA	NA	NA	NA	N	Y	Y	N	Y	70%

## DISCUSSION

The purpose of this review of the scientific literature is to investigate whether the Finite Element Analysis (FEA) can Influence of Variation in Dental Implant Dimensions on Peri-implant Bone Stress/strain distribution and how it will be useful to evaluate distribution of Stress/strain in periapical bone to calculate the Survival rate of implant. This investigation was carried out with the intention of determining whether this method should be utilized. Comparing the journals has been difficult because of variations in methodology. Nevertheless, this investigation has provided us with a comprehensive view of the outcomes that were achieved by the FEA technique as well as the applications of implantology.

Bone maintenance around dental implants is affected by several factors, including the implant and prosthesis materials, the nature of the loading, the direction and magnitude of force (axial or non-axial), and the quality and quantity of bone in the surrounding area. In the present review, Majority of studies used either cortical or cancellous or both layers to analyze the stress using FEA analysis. However, there was variation found in the quality analysis of bone. Researchers have previously zeroed in on cortical bone stress levels.^45^ This demonstrates, among other things, that the cortical bone is more susceptible to stresses than the trabecular bone due to its higher modulus of elasticity.

In the simulations, it was found that the cervical region of the short implants experienced a greater stress concentration than the rest of the implant^28^ Studies by several authors show that the values and distribution of stress at the cancellous bone-implant interface are primarily influenced by implant length, while the maximum implant diameter appears to affect stress peaks in the cortical bone but not in the trabecular region.^35^ In this analysis, we compare four common implant designs - threaded, tapered, Straumann, and screw vent tapered-along with their corresponding implant lengths, diameters, and types of loads (five studies compared axial, oblique, and lateral loads to comprehend FEA analysis).

**Fig 1:**
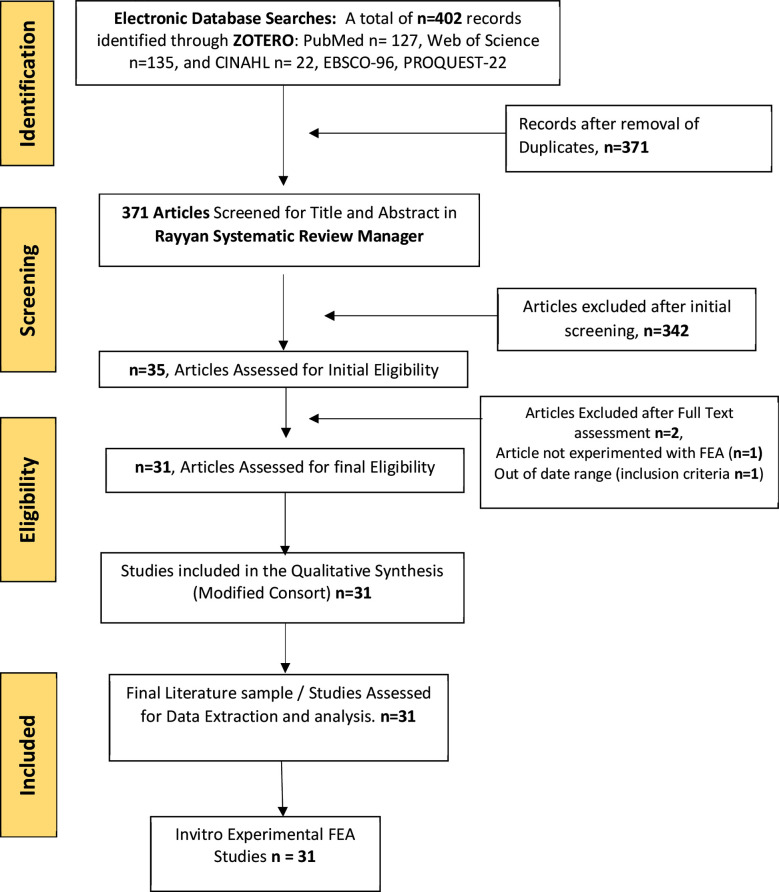
Finite Element Analysis (FEA) for Influence of Variation in Dental Implant Dimensions (Length and Diameter) on Peri-implant Bone Stress/strain distribution: Systematic Review PRISMA Flow Chart

The majority of studies used 100 to 200N of load for axial and obliques stress while Lu YJ et al^34^ used 50N stress for lateral load. Toniollo MB et al^25^ found that when looking at the results of the simulations, the vestibular side of the models experience a greater concentration of stress due to the oblique loads applied to them. This holds true for the abutments as well as the implants. Occlusal loading from an angle has also been linked to cortical bone overload in the neck region where compression is greatest.^32^ Stresses were concentrated more strongly on the abutments of the 13 mm and 11 mm implants than on the abutments of the 5 mm implants, according to the author, who hypothesizes that this is because the 0.8 mm high abutments have a larger cross-sectional area.^25^ Several authors have hypothesized that cortical bone stress can be reduced by increasing the implant’s length and diameter.^32^

Because longer implants have a greater surface area, it was also mentioned that it has been hypothesized that the stress levels are lower on longer implants for a given applied load. This was mentioned in conjunction with the fact that the length of the surface area increases.^22^ However, the findings of the simulation have shown that the influence of implant diameter and length on the stress state of the bone that is adjacent to the implant is not the only factor that needs to be considered when determining whether or not particular implant dimensions can be used in a particular clinical scenario. The comparison of the von Mises stress at the critical point of cortical bone with the ultimate strength for bone tissue is what determines the dimensions of the implant.^32^,^35^

One author investigated the relationship between stress and crown height and found that greater crown height, in conjunction with the shortest length of implants, caused greater stresses at the surface of the implant bodies. It has been reported that the purpose of the three-dimensional finite element analysis (FEA) was not to replicate the exact stresses found in vivo but rather to illustrate a possible difference in the way stresses are distributed on various bony ridges.^25^

Even though it was not possible to reproduce the conditions in vivo with a high degree of accuracy using finite element analysis, it was still possible to gain a better understanding of the biomechanical elements of short implants and how such stresses occur on the bone that is surrounding them.^46^ Short implants appear to be a workable solution in regions with reduced bone height, as Rokni et al.^47^ and Tawil et al.^48^ reached the same conclusion in 2005 and 2006, respectively. When equivalent stresses were compared, it was found that the stress was consolidated in the neck of the implant and the surface of the peri-implant bone, while the stress distribution inside the implant was approximately 6.0 MPa.

Author^4^ reported that increasing the thickness of the implant helps in reduction of stress more often than increasing the length. From the data analyzed here, we can conclude that the highest value of Von Mises stress in the bone around the implant decreases for screw-vent tapered and parallel design implants but increases for all other implant designs and diameters.^24^,^28^ FEM has been shown to increase the risk of bone resorption in the implant’s neck region in multiple publications. The von Mises strain values for the trabecular bone are consistently higher than those for the cortical bone throughout all the simulation runs. As the diameter of the implant grew, the highest value of the Von Mises equivalent stress was reported to decrease by Kong L et al.^21^ of the authors.^22^,^23^

Because it has been shown that spreading out the same force over a wider area result in lower stresses, screw-shaped implants were used. Implant threads separate the axial load into a component that is parallel to the thread plane and another component that is perpendicular to the thread plane. Therefore, the load transfer characteristics under normal masticatory forces and extreme load levels, such as those experienced during parafunction, were evaluated by measuring axial and off-axial loads.^49^ Any implant style, regardless of its superstructure’s composition or the force applied to it was found to produce peri-implant micro-strain values, with the standard diameter implant exhibiting the highest micro-strain values (3362.4 ±757.4) and the double mini-implant showing statistically significantly lesser mean micro=strains (801.6 ±251.4).^49^

Clinically, screw and cylinder implants are the most common, so FEA research compared the stress/strain in bone around these implants to that of a parallel-type implant. Studies using finite elements have shown that fewer stresses are placed on the bone around implants when they are larger in diameter. When it comes to biomechanics, larger implants are preferable because they can engage more bone and spread the load more evenly.^22^

Research on FEA and its application in in vivo studies is limited to studies conducted on animals and clinical cases with limited follow-up. The literature on this topic is extremely limited. Because of this, we are unable to conduct an objective analysis of the benefits provided by the method that was examined. There should be more interest in the product now that studies have begun on humans and animals in vivo with long-term follow-up to integrate bone compaction into the standard practice of implant surgery. Prospective cohorts and randomized controlled trials are two types of research that are necessary to carry out the necessary work that is required to fully establish the clinical outcomes of this method in a clinical setting.

## CONCLUSION

The most recent research suggests that the use of FEA showed promising results in understanding the stress distribution that surrounds the implant. The material of the dental implant and prosthesis, the type of loading, the direction and type of force, the quality of bone that surrounds the implant, and other factors all play a role in the maintenance of bone around dental implants. The cortical bone overloading may occur in the compressed part of its neck zone due to oblique occlusal loading, and a normal diameter implant showed the maximum micro-strain, while short implants seem to be a viable solution in areas with reduced bone height.

### Author Contributions:

**AJS, SFM:** Concept, design, methodology, formal analysis, and writing-original draft preparation.

**ASM** and **SFM:** Concept, design, critical review, are responsible for the accuracy or integrity of the work.

**SMRP, SKT:** Writing-review and editing, supervision.

**KKG, MASA, MSA:** Methodology, formal analysis, investigation, methodology.

**SKT, MAR:** Resources, writing-review and editing, project administration.

All authors have read the final manuscript.

## References

[1] Forna DA, Forna NC, Moldoveanu SAB (2020). Influence of implant dimensions in the resorbed and bone augmented mandible: A finite element study. Contemp Clin Dent.

[2] Baqain ZH, Moqbel WY, Sawair FA (2012). Early dental implant failure: Risk factors. Br J Oral Maxillofac Surg.

[3] Robau-Porrua A, Perez-Rodríguez Y, Soris-Rodríguez LM, Pérez-Acosta O, González JE (2020). The effect of diameter, length and elastic modulus of a dental implant on stress and strain levels in peri-implant bone: A 3D finite element analysis. Biomed Mater Eng.

[4] Naguib GH, Hashem ABH, Natto ZS, Abougazia AO, Mously HA, Hamed MT (2023). The Effect of Implant Length and Diameter on Stress Distribution of Tooth-Implant and Implant Supported Fixed Prostheses: An in Vitro Finite Element Analysis Study. J Oral Implantol.

[5] Cenkoglu BG, Balcioglu NB, Ozdemir T, Mijiritsky E (2019). The effect of the length and distribution of implants for fixed prosthetic reconstructions in the atrophic posterior maxilla: A finite element analysis. Materials.

[6] Didier P, Piotrowski B, Coz G Le, Joseph D, Bravetti P, Laheurte P (2020). Finite element analysis of the stress field in peri - implant bone: A parametric study of influencing parameters and their interactions for multi - objective optimization. Appl Sci (Switzerland).

[7] Chou HY, Muftu S, Bozkaya D (2010). Combined effects of implant insertion depth and alveolar bone quality on periimplant bone strain induced by a wide-diameter, short implant and a narrow-diameter, long implant. J Prosthetic Dent.

[8] Amine M, Guelzim Y, Benfaida S, Bennani A, Andoh A (2019). Short implants (5-8?mm) vs. long implants in augmented bone and their impact on peri-implant bone in maxilla and/or mandible: Systematic review. J Stomatol Oral Maxillofac Surg.

[9] Li T, Hu K, Cheng L, Ding Y, Ding Y, Shao J (2011). Optimum selection of the dental implant diameter and length in the posterior mandible with poor bone quality - A 3D finite element analysis. Appl Math Model.

[10] Brunski J (1988). Biomechanics of oral implants: future research directions. J Dent Educ.

[11] Shinya A, Ishida Y, Miura D, Shinya A (2021). The Effect of Implant Length and Diameter on Stress Distribution around Single Implant Placement in 3D Posterior Mandibular FE Model Directly Constructed Form In Vivo CT. Materials (1996-1944).

[12] Bhattacharjee B, Saneja R, Singh A, Dubey PK, Bhatnagar A (2022). Peri-implant stress distribution assessment of various attachment systems for implant supported overdenture prosthesis by finite element analysis –A systematic review. J Oral Biol Craniofac Res.

[13] Chaware S, Thakkar S (2020). A systematic review and meta-analysis of the attachments used in implant-supported overdentures. J Indian Prosthodont Soc.

[14] Dai C, Yang L, Guo L, Wang F, Gou J, Deng Z (2015). Construction of finite element model and stress analysis of anterior cruciate ligament tibial insertion. Pak J Med Sci.

[15] Okumura N, Stegaroiu R, Kitamura E, Kurokawa K, Nomura S (2010). Influence of maxillary cortical bone thickness, implant design and implant diameter on stress around implants: A three-dimensional finite element analysis. J Prosthodont Res.

[16] Zhou JJ, Zhao M, Yan YB, Lei W, Lv RF, Zhu ZY (2014). Finite element analysis of a bone healing model: 1-year follow-up after internal fixation surgery for femoral fracture. Pak J Med Sci.

[17] Liu S, Liu Y, Xu J, Rong Q, Pan S (2014). Influence of occlusal contact and cusp inclination on the biomechanical character of a maxillary premolar: A finite element analysis. J Prosthet Dent.

[18] Zhang C, Chang M, Zhang R, Tang S (2021). Biomechanical effects of osteoporosis on adjacent segments after posterior lumbar interbody fusion: A finite element study. Pak J Med Sci.

[19] Verri FR, Pellizzer EP, Rocha EP, Pereira JA (2007). Influence of length and diameter of implants associated with distal extension removable partial dentures. Implant Dent.

[20] Huang HL, Hsu JT, Fuh LJ, Tu MG, Ko CC, Shen YW (2008). Bone stress and interfacial sliding analysis of implant designs on an immediately loaded maxillary implant: A non-linear finite element study. J Dent.

[21] Kong L, Gu Z, Hu K, Zhou H, Liu Y, Liu B (2009). Optimization of the implant diameter and length in type B/2 bone for improved biomechanical properties: A three-dimensional finite element analysis. Adv Eng Softw.

[22] Mohammed Ibrahim M, Thulasingam C, Nasser KSGA, Balaji V, Rajakumar M, Rupkumar P (2011). Evaluation of design parameters of dental implant shape, diameter and length on stress distribution: A finite element analysis. J Indian Prosthodont Soc.

[23] Chatzigianni A, Keilig L, Duschner H, Götz H, Eliades T, Bourauel C (2011). Comparative analysis of numerical and experimental data of orthodontic mini-implants. Eur J Orthod.

[24] Ormianer Z, Amar A Ben, Duda M, Marku-Cohen S, Lewinstein I (2012). Stress and strain patterns of 1-piece and 2-piece implant systems in bone: A 3-dimensional finite element analysis. Implant Dent.

[25] Toniollo MB, Macedo AP, Rodrigues RCS, Ribeiro RF, Mattos M da GC de (2013). A three-dimensional finite element analysis of the stress distribution on morse taper implants surface. J Prosthodont Res.

[26] Saluja B, Alam M, Ravindranath T, Mubeen A, Nidhi A, Jyoti B (2012). Effect of length and diameter on stress distribution pattern of INDIDENT dental implants by finite element analysis. J Dent Implants.

[27] Bayraktar M, Gultekin BA, Yalcin S, Mijiritsky E (2013). Effect of crown to implant ratio and implant dimensions on periimplant stress of splinted implant-supported crowns: A finite element analysis. Implant Dent.

[28] Toniollo MB, Macedo AP, Rodrigues RCS, Ribeiro RF, de Mattos MDGC (2012). Three-dimensional finite element analysis of stress distribution on different bony ridges with different lengths of morse taper implants and prosthesis dimensions. J Craniofac Surg.

[29] Hasan I, Heinemann F, Bourauel C (2014). Biomechanical finite element analysis of self-tapping implants with different dimensions inserted in two bone qualitie. Biomedizinische Tech.

[30] Van Staden RC, Li X, Guan H, Johnson NW, Reher P, Loo YC (2014). A Finite Element Study of Short Dental Implants in the Posterior Maxilla. Int J Oral Maxillofac Implants.

[31] Ozyilmaz E Ahoedmbm (2014). Investigation of the effects of abutment and implant length on stability of short dental implants. Süleyman Demirel Üniversitesi Fen Bilimleri EnstitüsüDergisi.

[32] Demenko V, Linetskiy I, Nesvit K, Hubalkova H, Nesvit V, Shevchenko A (2014). Importance of diameter-to-length ratio in selecting dental implants: A methodological finite element study. Comput Methods Biomech Biomed Engin.

[33] Reddy Kr, Thumati P (2014). Influence of implant with different dimensions and designs in ideal stress distribution in bone for application in compromised situations: Analysis by three-dimensional finite element method. J Dent Implants.

[34] Lu Y, Chang S, Ye J, Ye Y, Yu Y (2015). Finite Element Analysis of Bone Stress around Micro-Implants of Different Diameters and Lengths with Application of a Single or Composite Torque Force. PLoS One.

[35] Abraham H, Philip J, Jain A, Venkatakrishnan C (2016). The effect of implant and abutment diameter on peri-implant bone stress: A three-dimensional finite element analysis. J Oral Res Rev.

[36] Eazhil R, Swaminathan S, Gunaseelan M, Kannan G, Alagesan C (2016). Impact of implant diameter and length on stress distribution in osseointegrated implants: A 3D FEA study. J Int Soc Prev Community Dent.

[37] Jomjunyong K (2017). Stress distribution of various designs of prostheses on short implants or standard implants in posterior maxilla: a three dimensional finite element analysis. Oral Implantol (Rome).

[38] Demenko V, Linetskiy I, Linetska L, Yefremov O (2019). Load-carrying capacity of short implants in edentulous posterior maxilla: A finite element study. Med Eng Phys.

[39] Raaj G, Manimaran P, Kumar CD, Sadan DS, Abirami M (2019). Comparative Evaluation of Implant Designs: Influence of Diameter, Length, and Taper on Stress and Strain in the Mandibular Segment--A Three- Dimensional Finite Element Analysis. J Pharm Bioallied Sci.

[40] Niroomand MR, Arabbeiki M (2020). Effect of the dimensions of implant body and thread on bone resorption and stability in trapezoidal threaded dental implants: a sensitivity analysis and optimization. Comput Methods Biomech Biomed Engin.

[41] Sheikhan E, Kadkhodazadeh M, Amid R, Lafzi A (2022). Interactive Effects of Five Dental Implant Design Parameters on the Peak Strains at the Interfacial Bone: A Finite Element Study. Int J Oral Maxillofac Implants.

[42] Lee H, Jo M, Sailer I, Noh G (2022). Effects of implant diameter, implant-abutment connection type, and bone density on the biomechanical stability of implant components and bone: A finite element analysis study. J Prosthet Dent.

[43] Gialain IO, da Silva LF, Takano MKG, Ballester YR, Roscoe GM, Meira BCJ (2022). Peri-implant bone resorption risk of anterior maxilla narrow single implants: a finite-element analysis. Biomater Investig Dent.

[44] Anitua E, de Ibarra NLS, Martín IM, Rotaeche LS (2022). Influence of Implant Tilting and Length on the Biomechanics of Single-Tooth Restoration: A Finite Element Analysis in Atrophic Mandible. Dent J (Basel).

[45] Weinberg LA KB (1995). A comparison of implant/prosthesis loading with four clinical variables. Int J Prosthodontics.

[46] Tawil P TG (2009). Short implants in deficient posterior jaws: current knowledge. Implant Dent.

[47] Rokni S TRWPPMAADD (2005). An assessment of crown-to-root ratios with short sintered porous-surfaced implants supporting prostheses in partially edentulous patients. Int J Oral Maxillofac Implants.

[48] Tawil G ANYR (2006). Influence of prosthetic parameters on the survival and complication rates of short implants. Int J Oral Maxillofac Implants.

[49] Kheiralla LS, Younis JF (2014). Peri-implant biomechanical responses to standard, short-wide, and mini implants supporting single crowns under axial and off-axial loading (an in vitro study). J Oral Implantol.

